# Optimal-Foraging Predator Favors Commensalistic Batesian Mimicry

**DOI:** 10.1371/journal.pone.0003411

**Published:** 2008-10-15

**Authors:** Atsushi Honma, Koh-ichi Takakura, Takayoshi Nishida

**Affiliations:** 1 Laboratory of Insect Ecology, Graduate School of Agriculture, Kyoto University, Kyoto, Japan; 2 Osaka City Institute of Public Health and Environmental Science, Osaka, Japan; University of Edinburgh, United Kingdom

## Abstract

**Background:**

Mimicry, in which one prey species (the Mimic) imitates the aposematic signals of another prey (the Model) to deceive their predators, has attracted the general interest of evolutionary biologists. Predator psychology, especially how the predator learns and forgets, has recently been recognized as an important factor in a predator–prey system. This idea is supported by both theoretical and experimental evidence, but is also the source of a good deal of controversy because of its novel prediction that in a Model/Mimic relationship even a moderately unpalatable Mimic increases the risk of the Model (quasi-Batesian mimicry).

**Methodology/Principal Findings:**

We developed a psychology-based Monte Carlo model simulation of mimicry that incorporates a “Pavlovian” predator that practices an optimal foraging strategy, and examined how various ecological and psychological factors affect the relationships between a Model prey species and its Mimic. The behavior of the predator in our model is consistent with that reported by experimental studies, but our simulation's predictions differed markedly from those of previous models of mimicry because a more abundant Mimic did not increase the predation risk of the Model when alternative prey were abundant. Moreover, a quasi-Batesian relationship emerges only when no or very few alternative prey items were available. Therefore, the availability of alternative prey rather than the precise method of predator learning critically determines the relationship between Model and Mimic. Moreover, the predation risk to the Model and Mimic is determined by the absolute density of the Model rather than by its density relative to that of the Mimic.

**Conclusions/Significance:**

Although these predictions are counterintuitive, they can explain various kinds of data that have been offered in support of competitive theories. Our model results suggest that to understand mimicry in nature it is important to consider the likely presence of alternative prey and the possibility that predation pressure is not constant.

## Introduction

Ever since the phenomenon of mimicry was first described [1], [2], it has drawn a great deal of attention, and it has been intensively studied as an example of Darwinian (co)evolution [3]–[8]. In Batesian mimicry, a palatable species (the Mimic) benefits from its resemblance to an unpalatable species (the Model), whose aposematic signal is therefore degraded in time; in this way, it is a parasitic relationship [1], [5], [8]. Müllerian mimicry involves two or more sympatric aposematic species, known as co-Mimics, that share the same or a similar warning pattern [2], [5], [8]. All of the unpalatable co-Mimic species benefit because they share the mortality costs of the predator learning process [2], [reprinted in 9]. Many studies have suggested that the evolutionary consequences of these two types of mimicry are distinct [5], [8], [10], [11]. In Batesian mimicry, a more abundant Mimic is expected to increase the predator attack rate on the Mimic as well as on the Model (negative frequency-dependent selection) and promote polymorphism in the Mimic, because an increase in the number of a certain type of Mimic is expected to decrease the fitness of that mimic [6], [10], [11]. In contrast, in Müllerian mimicry, the existence of co-Mimics is expected to reduce the per capita attack rate on both species, leading to number- or frequency-dependent selection and promoting monomorphism in the co-Mimics [6], [10], [11]. However, in some well-known examples of Müllerian mimicry, the co-Mimics are spectacularly polymorphic [12].

Huheey [13]–[15] proposed a mathematical model that challenged this classical Batesian–Müllerian dichotomy. Called the encounter-memory approach [8], the model assumes extraordinarily simple behavior on the part of the predator: the predator avoids both Models and Mimics following the attack on the Model, for subsequent n−1 encounters the number of which ( = n) positively correlates with the Model's unpalatability [13]. The model still yields results that agree very well with the experimental evidence [14 for a review]. However, the model has generated fierce criticism [16], [17] because of its radical prediction that mutualistic Müllerian relationships never occur. Instead, this model predicts that a less-defended co-Mimics always increases the attack probability on a more-defended co-Mimic and that the relationship can be neutral at best, when the unpalatability of the co-Mimics is identical [15]. The main cause of this counter intuitive prediction was claimed to be the encounter-based memory parameter of a predator: memories of a predator must change in a time-dependent manner [16], [17].

Speed [18] developed a time-based Monte Carlo predator model that incorporated psychology-based rules to describe learning, memory, and motivation of predators [19] to analyze the effects of the behavior and experience of an individual “Pavlovian” predator on the probability that it would attack a prey item. This succeeded in ‘rescuing’ mutualistic Müllerian mimicry: when both Müllerian co-Mimics are almost equally well defended, their relationship is mutualistic. In contrast to classical Müllerian mimicry, however, if one co-Mimic is less well defended then the protection of the better-defended one is diluted. This type of mimicry is called quasi-Batesian [18], [20] because even though both species are defended, the weakly defended species exploits the better-defended one in a parasitic, Batesian manner [18], [20]. Support has been growing for this approach, because a quasi-Batesian relationship can explain the observed polymorphism of co-Mimics in nature [2], [18 *cf*. 7], [Bibr pone.0003411-Mallet1] [21]. However, this quasi-Batesian relationship, where even a defended Mimic raises the predation risk of its Model, would seem counterintuitive, and the prediction has stirred new controversy as to whether it really occurs in nature [7], [9], [21].

The primary difference between the classical view of mimicry [2], [7], [9], [21] and the more recent challenges to this classical view [18], [20], [22], [23] is how they define unpalatability. In the former, a prey species always reduces its probability of being attacked by a predator by being unpalatable, and the probability thus approaches 0 [7] ( = so called “zero asymptote”, but not necessarily becoming zero (Jim Mallet, personal communication). This assumption naturally leads to the conclusion that although the benefits of mimicry may be greater for the less unpalatable species [21], mimicry between defended species is mutually beneficial even if large discrepancies exist between their defense levels [7], [9]. This assumption is valid when the co-Mimics have different population densities, a concept known as the natural history number-dependent view, because the protection of the co-Mimics depends on the combination of their unpalatability and abundance [2], [7], [21]. In the models challenging the classical view, a Pavlovian predator is assumed to attack prey with a fixed, non-zero asymptotic attack probability, after learning during a given time interval [18], [20], [23], whose value depends on the level of prey unpalatability. Referring to several examples in experimental studies [24]–[29], Speed [23] suggested that a stable attack number greater than 0 on a defended prey is rather common. In fact, bird attacks on apparently defended prey have been documented in nature [30], [31]. However, this assumption of a fixed, non-zero asymptotic attack probability has been criticized because it predicts that the number of prey attacked increases in direct proportion to the population size of the unpalatable prey [7], [9], [21]. Moreover, an experimental study demonstrated that a higher density of unpalatable prey reduced the proportion of prey being attacked [32], although the model predicts it to be fixed. It has also been argued that a quasi-Batesian relationship [18], [20] relies on an assumption that the attack probability can reach an asymptote at a value intermediate between 1 and 0 [7], [Bibr pone.0003411-Joron1] [9 *cf*. 23]; in this case, the less unpalatable species raises the attack probability on the more unpalatable species.

We propose here a mimicry model in which the Pavlovian predator system [18], [20] is expanded by including alternative prey (other than the Model, the Mimic, or co-Mimics) and a predator that follows optimal foraging strategy ( = a Darwinian predator), because the existence of alternative prey to the aposematic prey species has a significant effect on mimicry [33]–[35]. Predator behavior predicted by this model is consistent with the predictions of both Batesian–Müllerian mimicry theory and the theories that have challenged the classical view, depending on the availability of alternative prey. The probability of attack on unpalatable prey can approach 0 because an optimal forager excludes such prey from its diet when sufficient alternative prey are available. On the other hand, a non-zero asymptote is possible if alternative prey is rare. Moreover, a forgetful predator should occasionally attack unpalatable prey even after the attack probability has reached 0, with the result that the attack probability is never fixed at zero.

In mimicry studies, it seems reasonable to suppose that predator psychology should be taken into account because predators are the main selective agents that drive the evolution of the traits of the Model/Mimic species and the relationships between them [18], [20], [36], [37]. In previous models that consider predator behavior or psychology [18], [20], however, individual predators can choose only between models and mimics. Optimal foraging theory successfully predicts animal-foraging behavior according to some simple rules [38], [39] whereby individual predators choose their optimal diet menu. Incorporation of optimal foraging may lead to novel predictions about the relationships between Models and Mimics [40]–[42], but no theoretical framework exists that takes into account both optimal foraging and predator psychology [18], [20].

We developed a simulation model in which a psychologically based Monte Carlo predator [18], [20] behaves according to optimal foraging theory [38], [39], and examined how the introduction of alternative prey affected Model–Mimic relationships, and whether these relationships depended on the relative or absolute density of the Model species.

## Methods

### The simulation model

#### Behavior of a Darwinian predator

We constructed a simulation model describing predator behavior to examine the effect of incorporating an optimal diet choice strategy into Speed's Pavlovian predator model [18], [20] on the predicted relationships between Models and Mimics. This model approximates a predator that learns the value of a novel, aposematic prey from its foraging experience within a given locality and season.

The model includes two aposematic prey species: a highly unpalatable Model and a Mimic that is either palatable (Batesian) or less unpalatable (Müllerian). We defined palatability in accordance with Speed's predator psychology model [18], [20]. We also assumed perfect mimicry; that is, the two species are identical in appearance. Our model does not address the evolutionary dynamics of the aposematic traits of the Model and Mimic. We compared the risk of predation on the Model in the perfect mimicry condition and the risk in the condition where the Mimic has so different a color that the predator never generalizes it with the Model ( = the null model), and thus the “Mimic” is only a part of predator's daily diet. This comparison allows us to estimate the maximum load on the Model: the risk of predation on the Model is expected to be highest when the predator cannot discriminate these prey. The direction and value of the load drive the evolution of the Model's traits. The predator encounters Models and Mimics according to their respective population densities and the number of encounters with Model is independent of the density of Mimic (and vice versa), but the probability of an encounter leading to an attack is equal for both Models and Mimics because the predator cannot distinguish between them before attacking.

In many previous simulation models, predators repeatedly learn and forget the value of the prey based on experience, and the estimated prey value directly determines the motivation of the predator to attack the prey [18]–[20]. In our model, the predator estimates the prey value similarly, but then compares it to the value of an alternative diet, which is estimated independently of the aposematic species. If the inclusion of either the Model or Mimic into the predator's diet lowers its foraging efficiency, then the predator chooses not to attack, consistent with an optimal diet choice strategy [38].

Therefore, the probability of an attack on the aposematic prey depends on the following three factors: prey palatability, estimated by feeding experience (learning); time since the last feeding experience (forgetting); and the availability of alternative prey (decision making). We modeled each of these factors as shown below.

##### Learning prey value

We assume that the predator estimates the value of each of the different prey species according to a simple Pavlovian learning algorithm [18], [43]–[45]:
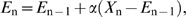
(1)where *E*_n_ is the estimated value of a prey item after the predator has experienced *n* feeding trials, *α* (0≤*α*≤1) is the learning rate of the predator, and *X*_n_ (0≤*X*_n_≤1) denotes the actual value of the prey item the predator encountered at the n^th^ feeding trial. This palatability valuable *X* should reflect fitness value because it is expected that in most cases preference corresponds to performance in the long term. As in the predator psychology model [18], palatable prey values range from 0.5 to 1.0, and unpalatable prey values from 0 to 0.5, with a neutral palatability of 0.5. The default value of the Model (*X*_mo_) was set to 0.2, while that of the Mimic (*X*_mi_) was varied between 0 and 1.0. When the predator attacks a Model, the value of the Model is substituted for *X*_n_ in Exp. 1. Similarly, when the predator attacks a Mimic, *X*_mi_ is substituted for *X*_n_.

##### Forgetting the prey value

Forgetting can be defined as the reversal of learning over time [18], in which memories become more difficult to retrieve with passing time [46]–[50]. The change in the estimated value of prey with the Model/Mimic signal caused by forgetting is defined by the following algorithm (as in [18]):
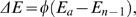
(2)where *φ* is the forgetting parameter (default value, 0.02), as in [18]. *E_a_* is the asymptotic value toward which the estimated value of the prey returns as a result of forgetting. This value was assumed to be 1 in all simulations, but the result is qualitatively insensitive to the specific value chosen.

##### Decision making

We assumed that the decision making of predator was affected by not only the value and abundance of the Models/Mimics but also those of alternative prey. When the reward value of the alternative prey is high and this prey is abundant, predators would likely choose not to include the Model/Mimic in their food menu. Conversely, when predators gain only a low reward value from alternative prey, they might be expected to attack the Model/Mimic. This model takes this decision making process into account. Predators estimate the value of Model/Mimic prey according to the results of their feeding trials, learning, and forgetting. Only when that estimated value is the same as or exceeds the mean value of the alternative prey species do they decide to attack the Model/Mimic. The attack probability, *P*_n_, is described by the following relations:
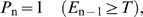
(3)

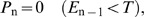
(4)where *T* corresponds to the mean reward per handling time of the alternative prey, which depends on the value of the left side of the following inequality from the theory of optimal diet choice [38], which describes the mean reward per searching and handling time.
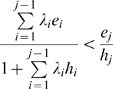
(5)where *e*, *h*, and *λ* denote units of net reward, handling time, and encountering rate of the prey. In this inequality, an optimally foraging predator which includes various kinds of prey in its diet in order of their profitability must decide whether or not to include the *j* ^th^ profitable prey in its diet: it should include the *j* ^th^ profitable prey if its profitability (the right side of the inequality) exceeds the mean reward of the first to the (j−1)^th^ profitable prey per searching and handling time (the left side). The estimated value of the prey, *E*, thus corresponds to the expression on the right side of Exp. 5. We assume, for the sake of simplicity, that all prey have the same handling time (*h*).

In our model, *T* is a thus threshold parameter and it was fixed during each simulation because the predator is expected to estimate the value of each prey discretely and its main diet is expected to be the alternative prey. Independent runs were performed with *T* values ranging from 0.2 to 1.0. Although *T* is a function of both the availability and individual quality of the alternative prey, for the sake of simplicity, we tentatively assumed that the value of a highly palatable Mimic was equal to the mean value of the alternative prey; thus, *T* was reduced to being a function of availability of the alternative prey.

In any given time interval, a predator randomly encounters at most one individual (either the Model or Mimic). For example, when the density of the Model (*D*_mo_) and that of the Mimic (*D*_mi_) are 0.2 and 0.3 respectively, a predator encounters the Model approximately 20% of the time and the Mimic about 30% of the time, and it does not encounter either about 50% of the time. The predator attacks the prey according to the probability *P*_n_; after each attack, the estimated value (*E*) of the Model/Mimic complex is renewed (Exp. 1). Subsequently, regardless of attack behavior, the forgetting rule modifies this value (*ΔE* in Exp. 2).

To construct the simulation program, we used Object Pascal language (see the source code in Supporting Information S1), and stochastic events (encounters with the Model, Mimic, or no prey; attacking prey or not) were coded using the pseudorandom number generator Mersenne Twister [51]. We ran the simulation for 1000 time intervals for each of 5000 virtual predators under a variety of conditions. Our preliminary simulations showed that the virtual predators always reached equilibrium between learning and forgetting before the 1000^th^ time interval. We defined the predation risk to the Model as the mean of the proportion of attacked individuals among all Models that a predator encountered in each trial of 5000 replications for each set of parameters. The behaviors of the virtual predators were estimated by Monte Carlo simulations, similarly to previous studies [18]–[20].

#### Predator psychology vs. alternative prey

The effect of the availability of an alternative diet on the relationships between the Model and Mimic was the most important target of this study. The threshold parameter *T*, indicating the availability of the alternative diet, was changed from 0.2 to 1.0 in steps of 0.05, and changed in the predation risk to the Model was observed. We then ran simulations in which the Model density was fixed at 0.2 and the Mimic density was varied from 0 to 0.8 in steps of 0.1 to examine how the Model species was affected when the density of Mimics increased.

**Figure 1:**
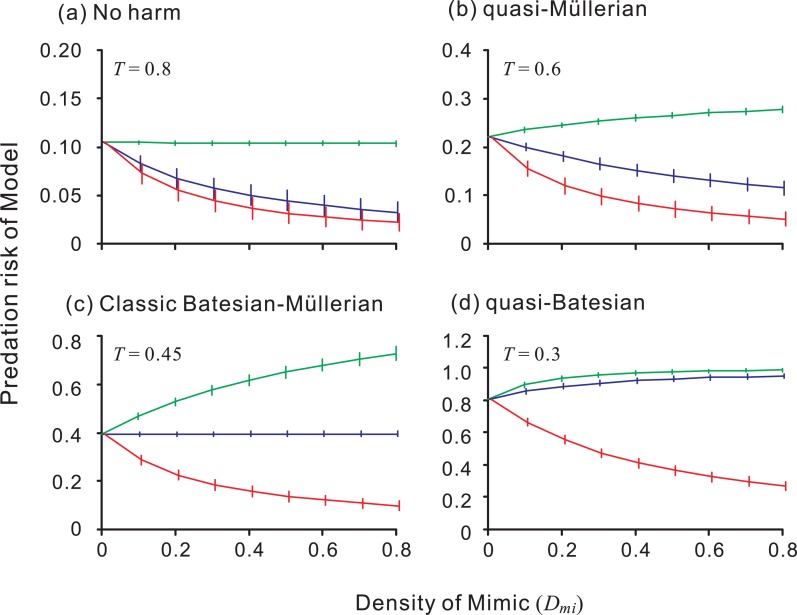
The four possible Model–Mimic relationships. Each point and error bar represent the mean and standard deviation of the replications. Blue, green, and red lines denote the Model's predation risk when a highly palatable Mimic (*X*_mi_ = 0.8), neutrally edible Mimic (*X*_mi_ = 0.5), or highly unpalatable Mimic (*X*_mi_ = 0.2), respectively, invades. (a) no harm: not even a highly palatable Mimic (*X*_mi_ = 0.8) increases the risk of predation on the Model; (b) quasi-Müllerian: a moderately palatable Mimic (0.5≤*X*_mi_<0.8) decreases the predation risk; (c) classical Batesian–Müllerian dichotomy: if the Mimic is palatable (*X*_mi_<0.5), it increases the predation risk of the Model, but if the Mimic is unpalatable (*X*_mi_>0.5), it decreases the predation risk. A neutrally edible Mimic (*X*_mi_ = 0.5) has no effect on the Model's predation risk; (d) quasi-Batesian: even a moderately defended Mimic (*X*_mi_<0.5) increases the predation risk. The negative effect of the Mimic on the Model increases in this order. The other simulation parameters, *w*, *ϕ* and density of Model (*D*_mo_), were fixed at 0.5, 0.02 and 0.2, respectively. The predator's decision-making rule was ‘all-or-nothing’. The threshold parameter *T* denotes the abundance of the alternative prey.

We classified the observed effects of the Mimic on the Model into four categories (Fig. 1): no harm, quasi-Müllerian, classical Batesian–Müllerian dichotomy, and quasi-Batesian. The criterion for no harm was that the predation risk to the Model did not increase even when a highly palatable Mimic coexisted. When the risk was increased by a highly palatable but decreased by a less palatable or unpalatable Mimic, the relationship was classified as quasi-Müllerian. A classical Batesian–Müllerian dichotomy was defined as when the risk to the Model increased as long as the Mimic was palatable, did not change if the Mimic was neutrally palatable, and decreased as long as the Mimic was unpalatable. Lastly, if the risk increased even when the Mimic was moderately unpalatable, then the relationship was classified as quasi-Batesian (Fig. 1). For this classification, we should calculate only three levels of predation risk on the model: risk_MO_ ( = the null model), when no Mimic existed (*D*_mi_ = 0 in Fig. 1); risk_HI_, when a highly palatable (*X*_mi_ = 0.8) Mimic coexisted at high density (*D*_mi_ = 0.7); and risk_NE_, when a neutrally edible (*X*_mi_ = 0.5) Mimic coexisted at high density (*D*_mi_ = 0.7). The criterion for no harm was risk_MO_≥risk_HI_. When risk_NE_<risk_MO_<risk_HI_, the system was classified as quasi-Müllerian. When the difference between risk_MO_ and risk_NE_ was not significant, the system was classified as the classical dichotomy. When risk_MO_<risk_NE_, the system was classified as quasi-Batesian. The risks were statistically compared using the Mann-Whitney *U*-test and the difference criterion was the significance level *P*<0.01.

Because predator psychology is also expected to affect the relationship between Model and Mimic, we assumed two different manners of learning by the predator: a fixed learning rate, where the learning rate *α* was held constant over each feeding trial and palatability was influenced by only the asymptote of the estimated value *E*_n_; and a variable learning rate *α*, in which the learning rate depended on prey palatability as well as on the asymptotic level of avoidance. To apply the latter rule, *α* in Expression 1 was multiplied by (0.5+|*X*_n_−0.5|). This modification causes the predator to learn more slowly when it encounters moderately (un)palatable prey than when it attacks highly (un)palatable prey; that is, the stronger the stimulus, the quicker the predator learns. Simulations were run with these two learning manners, and the learning rate of the last prey *α* was varied from 0.1 to 1.0 in steps of 0.05.

The forgetting parameter *φ* also affects the model's prediction, so we ran simulations in which *φ* was set at 0 (i.e., no forgetting over the whole season), as in [2], [7].

It has been demonstrated that the decision making of an optimally foraging predator is not exactly a matter of all or nothing, as predicted by optimal foraging theory [38] (and assumed in Exps. 3 and 4), but is probabilistic to some extent (e.g., [52]). We therefore examined two additional formulations of the attack probability *P*, besides that shown in Expression 3:
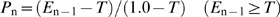
(6)where *P* increases linearly with increases in *E* (provided that *E*≥*T*) between *T* (*P* = 0) and 1.0 (*P* = 1.0), as in Speed's predator psychology model [18]; and
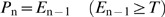
(7)where the shape of the *P* function is analogous to a dose-response [21]. In all formulations, *P* = 0 when *E*_n−1_<*T*.

#### Relative vs. absolute density of the Model species

We also examined whether the predation risk to the Model was affected by the absolute density of the Model (*D*_mo_) or by the relative densities of the Model and Mimic (*D*_mo_/*D*_mi_). We ran simulations with a fixed *D*_mo_/*D*_mi_ (1∶1) while *D*_mo_ varied (0.1–0.5). Then, we ran simulations in which *D*_mo_ was fixed at 0.2 and *D*_mo_/*D*_mi_ was varied from 0.1 to 0.5. For all of these simulations, the learning rate *α* and the forgetting parameter *φ*were set at 0.5 and 0.02, respectively. The predator's learning rate was fixed, and its decision making followed the ‘all-or-nothing rule’ (Exps. 3 and 4).

#### Accordance with predator behavior

The aforementioned assumptions about predator behavior were chosen so as to be consistent with laboratory evidence [53], especially when the predator's decision making rules were those described by expressions 6 (linear) and 7 (dose–response-like), in that learning rates were positively correlated with the mean prey defense level, the attack probability of each prey approached 0 (the asymptote) when the prey was sufficiently unpalatable, and the predator excluded a prey species from its optimal diet irrespective of its true value when its estimated value dropped below the threshold value (*T*) representing the value of alternative prey. Furthermore, after reaching the asymptotic level, the attack probability initially oscillated around the curve but in time stabilized at a value just above 0 as a result of forgetting. At that point, the predator began attacking the prey again, and learning again to avoid it. At the asymptotic level, the predator retained its learned aversion to more unpalatable prey for a longer period of time, and because the estimated value of the prey at the asymptote was negatively correlated with the mean defense level of the last prey taken, the period of attack avoidance (when *E*<*T*) was positively correlated with the mean level of prey defense. In addition, the number of prey attacked was negatively correlated with the mean level of prey defense.

## Results

### Predator psychology vs. alternative prey

**Figure 2:**
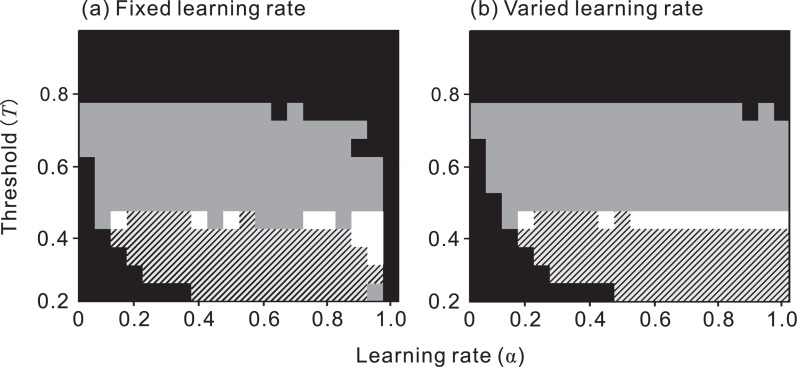
The effect of the learning rate (*α*) and rule of the predator learning, and the availability of alternative prey (*T*), on Model–Mimic relationships. (a) The predator learns the value of prey at the same rate *α* (fixed learning rate); (b) the predator learns more readily when it encounters highly palatable or unpalatable prey (varied learning rate). Black, no harm; gray, quasi-Müllerian; white, classical dichotomy; and hatched, quasi-Batesian. Huheey's rule [13], [14] in which only the last experience is remembered can be obtained by setting *α* equal to 1 following the fixed learning rule. The decision making rule of the predator is all-or-nothing (Exps. 3 and 4), *X*_mo_ = 0.2, *D*_mo_ = 0.2, *ϕ* = 0.02. Notice that predator's learning ability (*α*) and manner (fixed or varied *α*) have much less effect on Model-Mimic relationships than availability of alternatives (*T*).

The predator's learning ability, which is determined by *α*, had much less effect than the presence of alternative prey on Model–Mimic relationships, especially when at a high value of *T* (alternative prey are abundant: Fig. 2); the relationship was determined mainly by the value of the attack threshold (*T*) rather than by that of the learning rate (*α*). When alternative prey whose mean value is equal to that of a highly palatable Mimic (*X*_mi_ = 0.8) are abundant (*T*≥0.8), even the Mimic, which is expected to be included in the predator's optimal diet, does not harm the Model (no harm). When alternative prey are less abundant (0.8>*T*>0.4), a moderately palatable Mimic benefits the Model (quasi-Müllerian). The classic Batesian–Müllerian dichotomy emerges when alternative prey are rare (low *T*), and the area in which it emerges is small (Fig. 2). This result reflects the definition of the classical dichotomy, which makes it inevitable that the relationship will emerge at a certain point along the palatability spectrum of the Mimic; that is, the two categories of mimicry should switch instantaneously across a ‘knife-edge’ [16]. Quasi-Batesian mimicry emerges only when the availability of alternative prey is very low (very low *T*).

The predator's learning manner, fixed or varied (Fig. 2a and 2b, respectively), did not qualitatively affect the results, indicating that the fine details of predator learning had little effect on model prediction. Interestingly, a small *α* was beneficial to the Model: the parameter areas of no harm and quasi-Müllerian increase slightly.

While the other manner of forgetting (no forgetting: *φ* = 0) expanded the no harm area, no combinations of learning (fixed or varied), forgetting (forgetting or no forgetting), and decision making (‘linear’, Exp. 6, or dose–response, Exp. 7) rules produced a qualitatively different prediction from the default prediction shown in Fig. 2 (Supporting Information Fig. S1).

### Relative vs. absolute density of the Model

**Figure 3:**
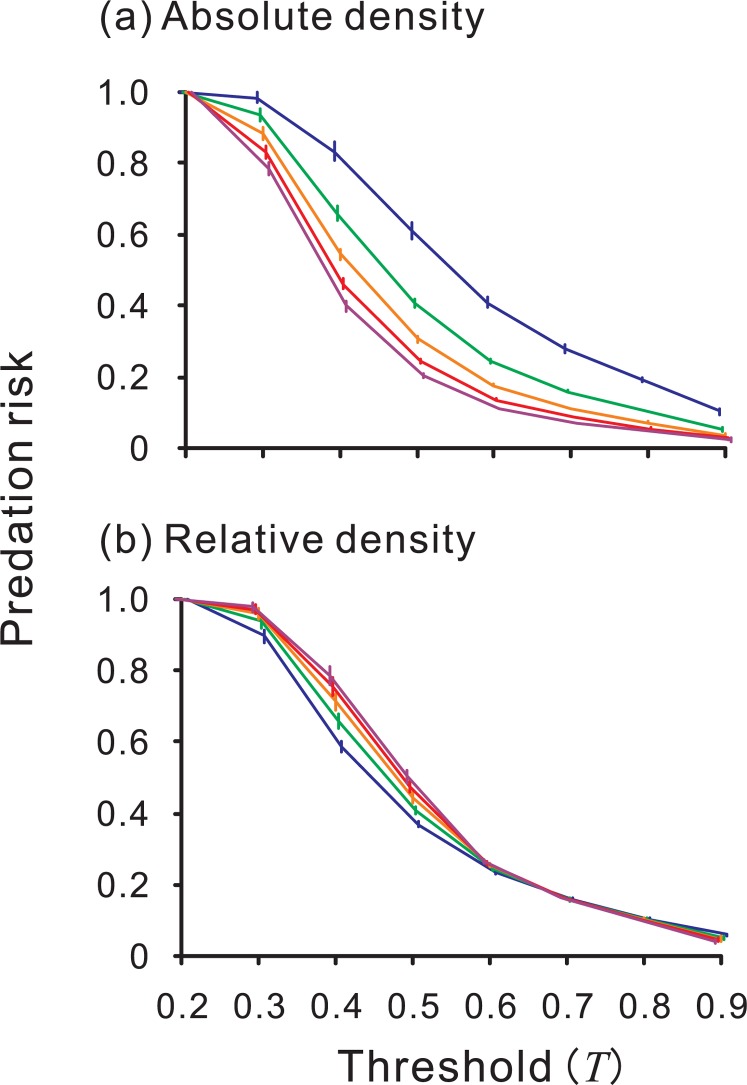
The effect of (a) absolute and (b) relative density of the Model on its predation risk. Each point and error bar represent the mean and standard deviation of the replications. *D*_mi_ = 0.1 (blue), 0.2 (green), 0.3 (orenge), 0.4 (red), or 0.5 (purple). (a) The density of both the Mimic and the Model is varied between 0.1 and 0.5 while keeping their ratio constant at 1.0. (b) The density of the Model is held constant at 0.2, while the density of the Mimic is varied between 0.1 and 0.5. *X*_mo_ = 0.2, *X*_mi_ = 0.8, *α* = 0.5, *φ* = 0.02. Notice that the risk of predation on the Model (and the Mimic) is influenced more by absolute density (*D*_mo_) than by relative density (*D*_mo_/*D*_mi_) of the Model.

The predation risk was influenced more by the absolute density of the Model (*D*_mo_) rather than by its density relative to that of the Mimic (*D*_mo_/*D*_mi_) (Figs. 3a and 3b): that is, the predation risk changed more when *D*_mo_ varied (Fig. 3a) than when *D*_mo_/*D*_mi_ varied (Fig. 3b), because as the density of the Model increased, the predator's attack probability (*P*) decreased, thus prolonging the period of ‘no attack’, which reduced the frequency with which a Mimic was attacked and raised the estimated value of the aposematic prey (*E*).

## Discussion

The basic structure of our simulation model is the same as that of the Monte Carlo predator system, which incorporates psychologically based rules for learning, memory, and motivation of predators [18], but because our model includes optimal foraging, it produces predictions that differ markedly from those produced by previous models. When alternative prey are abundant, as might be expected in nature, a Mimic whose value is the average of that of the predator's daily diet causes no harm to its Model; the predator's attack probability is determined mostly by the absolute density of the Model, not by its density relative to that of the Mimic. In the following sections, we discuss the general applicability of these predictions in the context of resolving several of the controversies regarding mimicry theory.

### Predator psychology vs. alternative prey

It is clear that a predator's learning ability affects the fitness of both the predator and the prey. An increase in the predator's ability to learn increases its foraging efficiency, which in turn increases or decreases the predation risk to the prey, depending on the predator's decisions. It has long been believed that a palatable Mimic harms its Model by interfering with the predator's aversion learning, thus degrading the predator's ability to estimate the Model's defense. Our simulations incorporating optimal foraging, however, predicted that predator psychology would have much less effect on the relationship between Models and Mimics than predicted by the predator psychology model [36] (Fig. 2 and Supporting Information Fig. S1). This counterintuitive prediction may explain why Huheey's mathematical model [13], [15], despite its extraordinary simplification of predator psychology, predicted the effect of Mimics on Models in several experiments with high accuracy [14] (also see *Relative vs. absolute density of the Model*). Indeed, in laboratory experiments, although predators did not behave as Huheey's mathematical model predicted, the model correctly predicted the frequency with which the Models and Mimics are attacked [14]. Mimicry systems are used by prey of predators of many different taxa, which suggests that mimetic relationships are possible for a wide variety of predator learning behaviors.

Our model predicted that a quasi-Batesian relationship could emerge only under very restricted conditions in which the availability of alternative prey was so low that the predator must either starve or eat unpalatable prey. This theoretical prediction is consistent with the predictions of dynamic optimization mathematical models of state-dependent foraging behavior by predators [40]–[42]. Although such conditions may be rare in nature, we interpret this prediction to agree with the predator psychology model proposed by Speed [18], [20]. Speed et al. [29], in an experiment in which wild birds were fed artificial prey in winter, demonstrated that a quasi-Batesian relationship could happen in nature: the presence of a moderately unpalatable prey (the Mimic) raised the attack rate on a more unpalatable prey (the Model). We argue that their result was critically dependent on the experimental setting: the experiment was conducted in winter when the availability of alternative prey was generally so low that even the unpalatable prey was of relatively high value. In this context, it is noteworthy to refer to the potential communication between signalers and receivers. For a signal to work, the receiver must have alternatives [54]; thus, an aposematic species should co-occur with an abundant palatable species.

Except in the case of a quasi-Batesian relationship, Müllerian polymorphism seems to be unstable because classical Müllerian mimicry theory predicts selection with a purifying effect, because an increase in the number of the aposematic prey decreases its risk of predation [10], [11]. Spatial and temporal heterogeneity can, however, explain mimetic polymorphisms in unpalatable species under mutualistic Müllerian mimicry [7], [12], [55], [56]. Even in mutual Müllerian relationships, the benefit received by co-Mimics is rarely symmetrical. As protection depends on a combination of unpalatability and abundance ([21], Appendix), less-defended and/or rare species can obtain more benefit by resembling a well-defended and/or more abundant species. Thus, a local population of a less-defended species should ‘adverge’ [10], [11], [57] to the most-defended and/or most-abundant species in each locality to obtain maximal benefit [21].

### Relative vs. absolute density of the Model

It has been widely suggested that the probability of attack by a predator on both a Model and on Batesian Mimics depends on not only the presence of the Model but also the relative abundance of Mimics and Models ([8] for a review). Our simulation, however, predicted that the absolute density of Models, rather than the relative abundance of Models and Mimics, determined the level of the protection of both Models and Mimics (Fig. 3).

**Figure 4:**
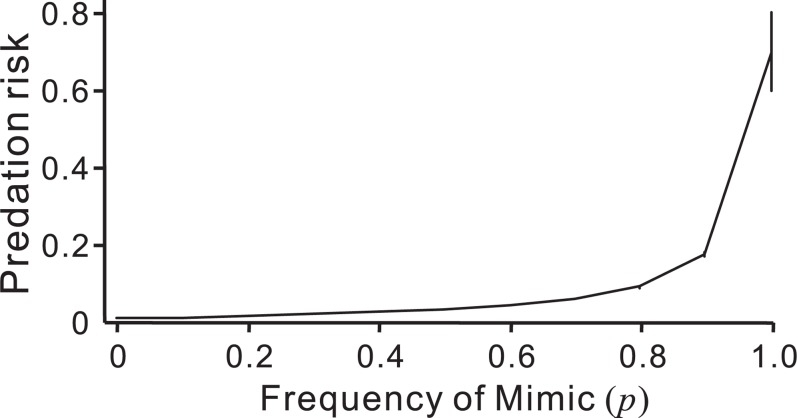
The result of a reciprocal frequency simulation. Each point and error bar denote the mean predation risk and its standard deviation of the replications. The abundance of the Mimic (p) relative to that of the Model is varied but the total number of prey is kept constant. *X*_mo_ = 0.2, *X*_mi_ = 0.8, *T* = 0.8, *α* = 0.5, *φ* = 0.02. Note that, in this reciprocal frequency treatment, increasing the ‘frequency’ of the Mimic raises the predation risk of the Model even in the condition in which the relationship would be no harm in Fig. 2.

The main, and seemingly counterintuitive, prediction of our simulation model is that the Mimic often does not harm the Model, even if the Mimic is sufficiently palatable to be included in the predator's optimal diet. This prediction seems inconsistent with experimental evidence showing that greater Mimic abundance always raises the attack probability on the Model [14], [58]–[60]. These experiments, however, adopted a ‘reciprocal frequency treatment’, called by Turner & Speed [61], in which the proportions of Models and Mimics were varied but the total number of prey was kept constant. As a result, an increase in Mimic availability dictated a decrease in Model availability. Under similar circumstances, our model predicted the same: a decrease in absolute density of the Model, rather than in the relative density of Models to Mimics, raised the attack probability on the Model (Fig. 4). The same can be said regarding an experiment that suggested that the less-defended co-Mimic raises the probability of attack on the well-defended co-Mimic in a quasi-Batesian manner [53]. To solve the problems that arise from the reciprocal frequency treatment, it is necessary to examine the effects of the Mimic on the Model by controlling both the frequency and density of the two species independently. In fact, a recent experimental study [62] reported that a palatable Mimic does not raise the attack probability of bird predators on the Mimic or its Model when the number of individuals of the Model is held constant and sufficient alternative prey are provided.

When Mimics invade a population of Models in nature, constant predation pressure can be realized by enhanced foraging activity that is exactly proportional to prey density. Although enhanced foraging activity may sometimes occur owing to Holling's numerical and Type I functional (when the density of the prey is relatively low) responses [63], [64] or apparent competition [65], [66], generally we do not expect predation pressure to increase in proportion to prey abundance. Moreover, for aposematism to be adaptive, the predator should be a generalist, but a generalist may not be sensitive to the density of minor components of its diet. If the Mimic outnumbers its Model, the predator may switch and begin attacking Mimics, although this may not often occur in nature.

**Figure 5:**
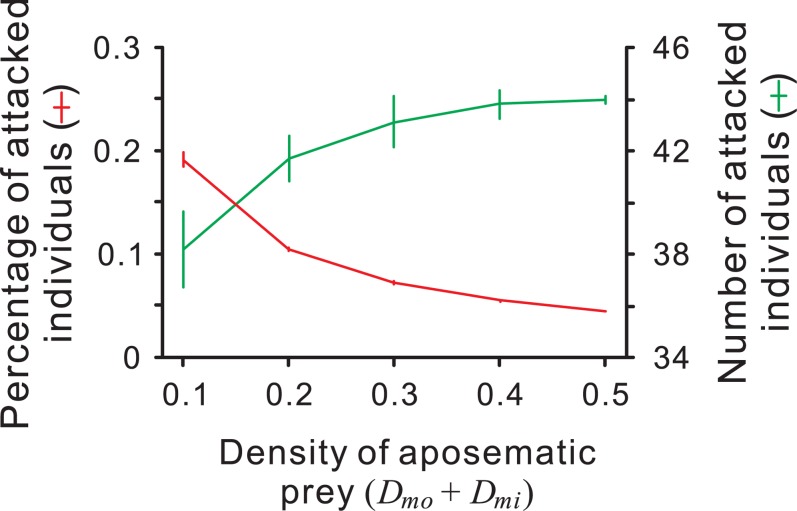
The result of a density multiplying simulation. The densities of the Mimic and the Model vary between 0 and 0.5 while their ratio remains constant (1∶1). Each point and error bar represent the mean and standard deviation of the replications. Red line and green line denote the mean percentage of prey and the mean number of prey being attacked, respectively. *X*_mo_ = 0.2, *X*_mi_ = 0.8, *T* = 0.8, *α* = 0.5, *ϕ* = 0.02. Increasing the density of the Model, the percentage of the prey attacked decreases while the number of them increases.

Moreover, our model can explain experimental evidence that previous theories could not completely explain. For example, Lindström et al. [32] demonstrated that captive great tits attacked unpalatable food items more frequently as the ‘population size’ the unpalatable food items increased, whereas the percentage of prey attacked decreased. Neither the traditional view (Müller's original theory [2], extended by Mallet [21]) nor Speed's predator psychology model [18], [20] predicted the result completely (see [23]): the former assumes that a fixed number of a prey species is killed during predator education [7], [21], and the latter predicts that a fixed percentage is killed. In our simulation, the percentage of prey items attacked (predation risk) decreased as the density of the Model increased (Fig. 5). However, the effect of the total density of Model and Mimic on the number of prey items attacked is predicted to be the opposite (Fig. 5).

### General discussion

Our model differs from previous models because it combines predator psychology and optimal decision making. It thus reveals two important factors which enable it to resolve discrepancies between the predictions of previous models and experimental and real-world observations. Exclusion of alternative prey has been predicted to play an important role in mimicry systems [33]–[35], and it leads predators to attack the possibly aposematic prey, thus diluting the protection provided by Model and Mimic aposematic patterns. However, if sufficient alternative prey items are available, this effect may dramatically decrease, because it is not necessary for the predators to certify the value of aposematic prey [67]. The other factor is the tacit assumption of constant predation pressure. According to the predictions of our simulation, it was the tacit assumption of constant predation pressure rather than its extraordinary simplified predator psychology that prevented Huheey's mathematical formula [13], [15] from predicting Müllerian mimicry (also see *Predator psychology vs. alternative prey*). In Huheey's model [13], [15], the predator's attack probability on aposematic prey did not increase even after attacks on the Mimic, although the model predicted that a Mimic would always raise the predation risk to the Model.

One controversy regarding the theory of Batesian mimicry centers around why Models do not simply evolve away from their Mimics [8]. The usual explanation is that mimicry may be a race that Models can never win because the relative success of rare Mimic mutants is much larger than that of Models [8], [11], [57]. Even if the Model could readily evolve away from its Mimics, the latter could evolve toward Models more quickly. In this case, a mimicry system may be a nonequilibrium coevolutionary race in which frequencies of aposematic traits of both participants change over time [68], [69]. Note, however, that a wide taxonomic range of predators show similar innate responses to common aposematic color patterns [5], suggesting that these aposematic signals have been rather stable over evolutionary time.

Our simulation suggests that the selection pressure on the Model to evolve away from palatable Mimics may be much less than is usually assumed. Mimicry rings, mimetic resemblances that often involve several species from many different families and orders [6], [10], may comprise one or a few Models that are mostly protected and abundant in each locality and other less-protected and rare Mimics (*cf*., [70], [71]). Even in the case of mimicry rings, our theory predicts that less-protected Mimics benefit Models, whereas unprotected Mimics simply take full advantage of the Model without harming it. Contrary to the traditional view (eg. [10], [11], [57]), mimicry rings may be evolutionarily stable.

It has been argued that the benefit of Batesian mimicry is negatively frequency-dependent on the Model/Mimic population because the presence of Mimics causes the attack rate to be higher on both species [1], [6], [10], [11]. According to this view, negative frequency-dependent selection may prevent Mimics from outnumbering Models [1], [6], [10], [11], and promote polymorphism in Mimics. In contrast, our model predicted the absence of such selection pressure only when alternative prey are abundant. While this prediction of our model may seem inconsistent with the occurrence of Batesian polymorphism, but such polymorphism can be explained by factors other than frequency-dependent predation. For example, polymorphism could still be beneficial as a bet-hedge because the densities of their Models in a certain locality should change seasonally, and then the predation pressure on each signaler may be reversed. Spatial and temporal heterogeneity also can explain Batesian polymorphisms [7], [12], [55], [56].

Our model provides a conceptual framework for mimicry that can explain the predictions of several mathematical models that were apparently inconsistent with experimental results. The relationships described by previous theories are predicted by our model to occur only when the availability of alternative prey is low (or the value of the Mimic is higher than that of alternative prey). Batesian Mimics have traditionally been viewed as having a parasitic relationship with their Models. Our model also suggests that Batesian mimicry is more often commensalistic than parasitic. This view may require a reconsideration of the simple Batesian–Müllerian dichotomy, along with the recent claim that the selective mechanisms of Müllerian mimicry may include the advergence of less-defended species toward the more-defended one [21]. As Nicholson [72] suggested, Batesian and Müllerian mimicry may be extreme types along a spectrum of deceptive resemblance rather than two separate phenomena.

## Supporting Information

Figure S1 Simulation results for 10 combinations of learning rules (fixed or variable), forgetting rules (forgetting or no-forgetting), and decision making rules (linear response, dose-response, or all-or-nothing) (two other combinations are shown in Fig. 2). (a–e) Fixed and (f–j) variable learning rate. (a, b, f, g) forgetting (*ϕ* = 0.02) and (c–e, h–j) no forgetting (*ϕ* = 0). (c, h) Linear, (a, d, f, i) dose-response, and (b, e, g, j) all-or-nothing responses. The color coding is the same as in Fig. 2. Changes in learning, forgetting and decision making rules, and their combinations do not effect the general result.
(0.55 MB EPS)

Supporting Information S1 This archive contains source code files of the simulation model used in the manuscript by Honma et al. (2008). For users of MS Windows, please compile honma2008.dpr with Borland Delphi. About free versions of Delphi, please refer to the following site. http://cc.codegear.com/free/delphi For users of ther Operating systems (Linux, FreeBSD, MacOS/OSX, OS/2), please compile honma2008.pas with Free Pascal. Free Pascal Compiler is freely downloadable from the following site. http://www.freepascal.org/ Contents 1. honma2008.dpr Pascal code file for Borland Delphi (ver.5 or later). 2. honma2008.pas Pascal code file for Free Pascal (ver.2 or later). 3. random.pas/randomfp.pas Pascal unit files coding the Mersenne Twister random generator. Random.pas for Delphi and randomfp.pas for Free Pascal. These source codes is based on that by H.Yamamoto. http://www.asahi-net.or.jp/jz6h-ymmt/download/MTmain.pas 4. params.txt Text file including parameters of the simulateion. This file is used in execution of the compiled binary.
(0.01 MB ZIP)
